# 10′(*Z*),13′(*E*)-Heptadecadienylhydroquinone Inhibits Swarming and Virulence Factors and Increases Polymyxin B Susceptibility in *Proteus mirabilis*

**DOI:** 10.1371/journal.pone.0045563

**Published:** 2012-09-20

**Authors:** Ming-Che Liu, Shwu-Bin Lin, Hsiung-Fei Chien, Won-Bo Wang, Yu-Han Yuan, Po-Ren Hsueh, Shwu-Jen Liaw

**Affiliations:** 1 Department and Graduate Institute of Clinical Laboratory Sciences and Medical Biotechnology, National Taiwan University, Taipei, Taiwan, Republic of China; 2 Graduate Institute of Microbiology, College of Medicine, National Taiwan University, Taipei, Taiwan, Republic of China; 3 Department of Surgery, National Taiwan University Hospital, Taipei, Taiwan, Republic of China; 4 Department of Laboratory Medicine, National Taiwan University Hospital, Taipei, Taiwan, Republic of China; University of Helsinki, Finland

## Abstract

In this study, we demonstrated that 10′(*Z*), 13′(*E*)-heptadecadienylhydroquinone (HQ17-2), isolated from the lacquer tree, could decrease swarming motility and hemolysin activity but increase polymyxin B (PB) susceptibilityof *Proteus mirabilis* which is intrinsically highly-resistant to PB. The increased PB susceptibility induced by HQ17-2 was also observed in clinical isolates and biofilm-grown cells. HQ17-2 could inhibit swarming in the wild-type and *rppA* mutant but not in the *rcsB* mutant, indicating that HQ17-2 inhibits swarming through the RcsB-dependent pathway, a two-component signaling pathway negatively regulating swarming and virulence factor expression. The inhibition of hemolysin activity by HQ17-2 is also mediated through the RcsB-dependent pathway, because HQ17-2 could not inhibit hemolysin activity in the *rcsB* mutant. Moreover, the finding that HQ17-2 inhibits the expression of *flhDC* gene in the wild-type and *rcsB-*complemented strain but not in the *rcsB* mutant supports the notion. By contrast, HQ17-2 could increase PB susceptibility in the wild-type and *rcsB* mutant but not in the *rppA* mutant, indicating that HQ17-2 increases PB susceptibility through the RppA-dependent pathway, a signaling pathway positively regulating PB resistance. In addition, HQ17-2 could inhibit the promoter activities of *rppA* and *pmrI*, a gene positively regulated by RppA and involved in PB resistance, in the wild-type but not in the *rppA* mutant. The inhibition of *rppA* and *pmrI* expression caused lipopolysaccharide purified from HQ17-2-treated cells to have higher affinity for PB. Altogether, this study uncovers new biological effects of HQ17-2 and provides evidence for the potential of HQ17-2 in clinical applications.

## Introduction

*Proteus mirabilis* is an important pathogen of the urinary tract, and is the primary infectious agent in patients with indwelling urinary catheters [1]. Several potential virulence factors may be responsible for the pathogenicity of *P. mirabilis*. Among them, flagella, necessary for swarming, are involved in establishing infection [1]. Haemolysin, which is cytotoxic for cultured urinary tract epithelial cells [2], has been shown to be correlated with the ability of bacteria to invade cells [3]. The ability of *P. mirabilis* to express virulence factors, such as haemolysin, and to invade urothelial cells, is coordinately regulated with swarming differentiation [3], [4], [5], [6].

Characterization of *Proteus* mutants has indicated that a substantial number of proteins, including FlhD_2_C_2_, RsbA (also known as RcsD) and RsmA, are involved in regulation of swarming and virulence factor expression [7], [8], [9], [10]. Among these regulatory proteins, RcsD has been shown to act as a negative regulator of swarming differentiation and virulence factor expression in *P. mirabilis* [5], [6], [10], [11]. In *Escherichia coli*, the RcsCDB (Rcs) signal transduction system consists of three proteins: the sensor RcsC, the cognate response regulator RcsB and the histidine-containing phosphotransfer protein RcsD [12]. It has been determined that the flow of phosphoryl groups through the Rcs phosphorelay components occurs as follows: RcsC
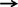
RcsD
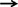
RcsB [13]. This Rcs system appears to be conserved in the family Enterobacteriaceae, and it is involved in controlling the transcription of a vast range of genes, such as those regulating flagellum synthesis, O-antigen chain length, and virulence [12], [13]. It is noteworthy that the Rcs system negatively regulates the transcription of the *flhDC* flagellar master switch in *E. coli*, *Salmonela* and *P. mirabilis* [14], [15], [16]. FlhD_2_C_2_ is necessary for swarming and hemolysin activity in *P. mirabilis* [7], [9], [14], [17]. During swarmer cell differentiation, the transcript levels of *flhDC* rise almost 50-fold; therefore, mutations in genes that regulate *flhDC* levels can have dramatic effects on swarming. For example, mutations in components of the RcsBCD phosphorelay which negatively regulates *flhDC* result in hyperswarming [5], [10], [14].

*P. mirabilis* is known to be naturally highly resistant to polymyxin B (PB), a kind of cationic antimicrobial peptides (CAPs). CAPs play an important role in host defense against microbial infection and are key effectors of the host innate immune response [18]. The ability of *P. mirabilis* to survive the killing action of CAPs is clearly important in the pathogenesis of urinary tract infections [19], [20], [21]. In gram-negative bacteria, CAPs, which have a net positive charge and an amphipathic structure, bind to the negatively charged residues of lipopolysaccharide (LPS) of the outer membrane and then can alter bacterial membrane integrity by solubilization or pore formation [22]. In *Salmonella*, a seven-gene operon (*pmrHFIJKLM*) is involved in an LPS modification [23], [24]. The gene products of the operon are necessary for the biosynthesis and addition of 4-aminoarabinose (Ara4N) to lipid A-a modification which contributes to a reduction in the net negative charge of LPS and consequently decreases attraction and binding of CAPs to the outer membrane [23], [24]. Previously, we identified an *rppA* gene which is located upstream of the *rppB* gene in *P. mirabilis* [25]. The *rppA* and *rppB* genes may encode a response regulator and a membrane sensor kinase, respectively, of a two-component system (TCS) in *P. mirabilis*. RppA directly controls the expression of a *pmrHFIJKLM* homologue, thus leading to PB resistance [25], [26]. Moreover, activated RppA can bind to the *rppAB* promoter and stimulate its own transcription [25].

Hydroquinone (HQ) is a well-known tyrosinase inhibitor and antimelanogenesis compound that has been used as an active ingredient in cosmetics and pharmaceuticals since the 1960s [27]. However, the use of HQ in cosmetics has been banned for the sake of safety [27]. Three hydroquinone derivatives have been isolated from the sap of the lacquer tree *Rhus succedanea*, 10′(*Z*)-heptadecenylhydroquinone (HQ17-1), 10′(*Z*),13′(*E*)-heptadecadienylhydroquinone (HQ17-2) and 10′(*Z*),13′(*E*),15′(*E*)-heptadecatrienylhydroquinone (HQ17-3) [28]. HQ17-1 was found to inhibit the activity of tyrosinase and to suppress melanin production in animal cells [29]. As tyrosinase activity accounts for postharvest browning of botanical products and animal skin melanogenesis, HQ17-1 could be useful for the preservation of these products or as a skin-whitening cosmetic. HQ17-3 exhibits anticancer activities by acting as a topoisomerase II poison [30]. All three hydroquinone derivatives have no significant cytotoxic effect on non-dividing cells, such as peripheral blood mononuclear cells, and differ in their cytotoxicity to various cell lines [28], [30] (our unpublished data), with HQ17-3 being the most toxic [28], [30]. The biological roles of HQ17-2 and its cytotoxicity to various cell lines are lacking and need to be explored.

Targeting bacterial virulence factors is now gaining interest as an alternative strategy to develop new types of anti-infective agents [31]. Two-component systems are often involved in the virulence of many pathogens, making them attractive targets for antimicrobial drug development [32]. In this study, we report that HQ17-2 can reduce virulence factor expression through the RcsB-dependent pathway and increase PB susceptibility by inhibiting the promoter activities of *rppA* and *pmrI* in *P. mirabilis*. To our knowledge, this is the first report demonstrating that a natural compound, HQ17-2, can reduce virulence factor expression and enhance efficacy of PB in *P. mirabilis*. This study not only uncovers new biological effects of HQ17-2 but also provides evidence for the potential of HQ17-2 in clinical applications.

## Materials and Methods

### Bacterial Strains, Plasmids, Reagents and Growth Conditions

The bacterial strains and plasmids used in this study are listed in Table 1. Bacteria were routinely cultured at 37°C in Luria-Bertani (LB) medium. The LSW^-^ agar was used to prevent the phenotypic expression of swarming motility [25], [33]. 10′(*Z*), 13′(*E*)-heptadecadienylhydroquinone (HQ17-2) is a new antioxidative compound isolated from the sap of *Rhus succedanea* [28]. The purification protocol was modified from that described previously [28]. An aliquot of the lacquer (10 g) was dissolved and mixed with 90 ml of 80% EtOH. The mixture was centrifuged (8000 *g*, 5 min). The supernatant was subsequently extracted with a 3-fold volume of ethyl acetate and centrifuged at 700 g for 20 min. The ethyl acetate layer was collected and vaccuum-dried. The residue was dissolved in 100% EtOH and subjected to HPLC (Chrom Tech, Inc., USA) analysis on a preparative C18 column, isocratic elution by 90% MeOH (5 ml/min), and a detector monitoring at OD_280_. The purified HQ17-2 was dissolved in EtOH, and the UV absorption spectrum was measured using a SpectraMax M5 Microplate Reader (Molecular Devices, USA). The purity was also checked by HPLC using an analytical C18 column, isocratic elution by 90% MeOH (1 ml/min), and a photodiode array detector (DAD 230, Chrom Tech, Inc.). The HQ17-2, with purity over 99%, was used for the following experiments. HQ17-2 at the concentration of 72.6 µM was used in the most experiments unless indicated specifically.

**Table 1:** Bacterial strains and plasmids used in this study.

Strain or plasmid	Genotype or relevant phenotype	Source or reference
*Proteus mirabilis*		
N2	Wild-type; Tc^r^	[25]
dA10	N2 derivative; *rppA*-knockout mutant; PB^s^ Km^r^	[25]
dA10c	dA10 containing pGEM-T Easy-*rppA*; *rppA*-complemented strain; Amp^r^	[25]
dIp	N2 derivative; *pmrI* knockout mutant; PB^s^ Km^r^	[26]
dIpc	dIp containing pACYC184-*pmrI*; *pmrI*-complemented strain; Cm^r^	[26]
rcsB	N2 derivative; *rcsB*-knockout mutant; Km^r^	This study
rcsBc	rcsB containing pACYC184-*rcsB*; *rcsB*-complemented strain; Cm^r^	This study
rcsBov	*rcsB-*overexpressed N2; *rcsB* (with its ribosome binding site) cloned into the high-copypGEM-T Easy with intact *rcsB* oriented behind *lac* promoter; Amp^r^	This study
CI1∼11	Highly resistant to PB	Clinical isolates
Plasmids		
pGEM-T Easy	High-copy TA cloning vector; Amp^r^	Promega
pUT/mini-Tn*5*(Km)	A suicide plasmid requiring Pir protein for replication and containing mini-Tn*5* cassettecontaining Km^r^ gene	[26]
pACYC184-*rppA-xylE*	pACYC184 containing *rppA* promoter sequence and *xylE* coding region; Cm^r^	This study
pACYC184-*pmrI-xylE*	pACYC184 containing *pmrI* promoter sequence and *xylE* coding region; Cm^r^	[26]
pACYC184-*flhDC-xylE*	pACYC184 containing *flhDC* promoter sequence and *xylE* coding region; Cm^r^	This study

### Swarming Assay

The swarming migration assays was performed as described previously [25], [34]. Briefly, an overnight bacterial culture (5 µl) was inoculated centrally onto the surface of dry LB swarming plates containing 2% (w/v) agar with or without HQ17-2, which were then incubated at 37°C. The swarming migration distance was measured by following swarm fronts of the bacterial cells at 1 h intervals.

### Measurement of Hemolysin Activity

An overnight LB culture of the bacteria was inoculated onto the surface of dry LB swarming plates with or without HQ17-2, which were then incubated at 37°C. Hemolysin activity was determined 2 h after inoculation and hourly thereafter. Preparation of cells for hemolysin assay and measurement of hemolysin activity were performed as described previously [34].

### Cell Invasion Assay

The cell invasion assay was performed as described by Jiang *et al*. [26]. An overnight *P. mirabilis* culture was diluted 100-fold and incubated for 3 h before the cell invasion assay was performed. In this study, HQ17-2 was included in the bacterial suspension during the 1.5 h infection period to investigate the effect of HQ17-2 on the cell invasion ability of *P. mirabilis*.

### Construction of *P. mirabilis rcsB* Mutant

For construction of the *rcsB* mutant, the primer pair, RcsB1/RcsB2 (Table 2), was used to amplify the central region of *rcsB* (*rcsB*c). The PCR product was cloned into pGEM-T Easy vector (Promega, USA) to form pGEM-*rcsB*c. The pGEM-*rcsB*c was then digested with *Xba*I (a site close to *rcsB*c fragment), and ligated with the Km-resistant cassette to form pGEM-*rcsB*c*/Km*. The *rcsBc/Km-*containing fragment was cut with *Sal*I*-Sph*I and ligated into *Sal*I*-Sph*I digested pUT vector to form pUT-*rcscB*c*/Km*. For inactivation of *rcsB* gene by homologous recombination, pUT-*rcsB*c*/Km* was transferred from *E. coli* S17-1 to *P. mirabilis* N2 by conjugation. Transconjugants were spread on LSW^-^ plates containing kanamycin (100 µg/ml) and tetracycline (12.5 µg/ml). Mutant candidates were screened by colony PCR and Southern blot hybridization was performed to confirm the mutant genotype. Results confirmed that a single-crossover event had occurred.

**Table 2:** Primers used in this study.

Primer	Sequence (5’ to 3’ )	Description
flhDC RT-F	CGCACATCAGCCTGCAAGT	For *flhDC* real-time PCR. Paired with “flhDC RT-R”
flhDC RT-R	GCAGGATTGGCGGAAAGTT	
16SrDNA RT-F	CACGCAGGCGGTCAATTAA	For 16S rDNA real-time PCR. Paired with “16SrDNA RT-R”
16SrDNA RT-R	GCCAACCAGTTTCAGATGCA	
flhDC promoterF	GGGTAGATTCGCTTATTAATTCTC	For *flhDC* reporter assay. Paired with “flhDC promoterR”
flhDC promoterR	CTCTTTACATCCCGTCCGAT	
pmr promoterF	CAACAATCGTTAGCTTTGCC	For *pmrI* reporter assay. Paired with “pmr promoterR”
pmr promoterR	GGAGAATGGAAGAAAGTGAC	
rppA promoterF	GCATTACTTTCTATGAGTTAACTCTTTGG	For *rppA* reporter assay. Paired with “rppA promoterR”
rppA promoterR	TTTCCCAATTGCAGATCGTC	
rcsB1	TGCCGATGATCATCCAATTG	For construction of the *rcsB-*knockout mutant. Paired with “rcsB2”
rcsB2	TCTAGAGGCATAGATAGGTCGGTG	
rcsBoverF	GGATCCGACAGTTGCTGT	For construction of the *rcsB-*overexpressed strain. Paired with “rcsBoverR”
rcsBoverR	ACGGGTTAGCTTTCTCCG	
rcsBcomR	GCATGCACGGGTTAGCTTTCTCCG	For construction of the *rcsB-*complemented strain. Paired with “rcsBoverF”

### Construction of *P. mirabilis rcsB*-overexpressed Strain

Full-length *rcsB* (with its ribosome binding site) was amplified by PCR using the primer pair rcsBoverF and rcsBoverR (Table 2) and cloned into pGEM-T Easy (Promega) with intact *rcsB* oriented behind *lac* promoter. The constructed plasmid was transformed into the wild-type *P. mirabilis* to generate the *rcsB*-overexpressed strain.

### Construction of *P. mirabilis rcsB*-complemented Strain

Full-length *rcsB* was amplified by PCR using the primer pair rcsBoverF and rcsBcomR (Table 2) and cloned into pGEM-T Easy (Promega) to generate pGrcsB. The DNA fragment containing full-length *rcsB* was excised from pGrcsB with *Bam*HI and *Sph*I. The DNA fragment was ligated into a *Bam*HI/*Sph*I-digested low-copy-number plasmid, pACYC184, to generate the *rcsB* complementation plasmid, pACYC184-*rcsB*. *rcsB* is thus driven by the promoter of the tetracycline-resistant gene in the pACYC184 plasmid. pACYC184-*rcsB* was then transformed into the *rcsB* mutant to generate the *rcsB*-complemented strain.

### Real-time RT-PCR

Overnight-LB cultures of wild-type *P. mirabilis*, the *rcsB* mutant and the *rcsB-*overexpressed and complemented strains were diluted to an optical density at 600 nm of 0.3–0.4, inoculated into LB broth without or with HQ17-2 (to examine its effect on the expression of *flhDC* when needed) and grown for 4 h at 37°C. Total RNA was extracted and real-time reverse transcription (RT)-PCR was performed as described [34] to monitor the expression of *flhDC* mRNAs using primer pairs listed in Table 2. The levels of *flhDC* mRNAs were normalized against 16S rRNAs.

### Reporter Assay

Reporter plasmids of *rppA* and *flhDC* were derived from the *pmrI-xylE* plasmid [26]. In brief, *pmrI* promoter region of the *pmrI-xylE* plasmid was replaced by the *rppA* or *flhDC* promoter region to produce the *rppA-* and *flhDC-xylE* reporter plasmids. The wild-type, the *rppA* mutant, the *rcsB* mutant or the *rcsB*-overexpressed strain of *P. mirabilis* was transformed with the respective reporter plasmid and grown overnight in LB broth containing 20 µg/ml chloramphenicol. The cultures were diluted 100-fold in the same medium with or without HQ17-2 and incubated at 37°C for 3 to 6 h. PB (1 µg/ml) was added simultaneously with HQ17-2 in the induced condition of the RppA
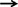
PmrI pathway. The XylE activity was measured as described previously [26].

### MIC Assay

In vitro determination of MIC for polymyxin B (Fluka, USA) was performed by the broth microdilution method according to the guidelines proposed by Clinical and Laboratory Standard Institute [35] in the presence or absence of HQ17-2. A stock solution of PB (40960 µg/ml) prepared in Mueller Hinton broth was added to 96-well microtiter plates in two-fold serial dilutions. Aliquots of bacterial culture (5 × 10^4^ CFU) were then dispensed into each well and incubated for 16–18 h. MICs for sodium dodecyl sulfate (SDS), gentamycin, kanamycin, streptomycin, ampicillin, tetracycline and ciprofloxacin was also determined. MIC was defined as the lowest concentration at which no visible growth occurred.

### Killing of Biofilm-grown *P. mirabilis* by PB

This experiment was performed as described by Lee *et al*. [36] with some modifications. An overnight culture of *P. mirabilis* N2 was diluted to 1.5 × 10^7^ CFU/ml and 10 µl of bacterial cultures was applied onto each of three 1 cm^2^ nitrocellulose (NC) membranes (Millipore, USA) separately placed on LSW^-^ agar plates. The plates were then incubated at 37°C to allow biofilm formation. Forty-eight hours later, the membranes containing biofilm-grown bacteria were then treated with 10 µl of a solution containing PB (10240 or 20480 µg/ml), a solution containing PB (10240 or 20480 µg/ml) plus 72.6 µM HQ17-2, or a solvent control. After incubation for 16 h, the bacteria from individual membrane were suspended in 1 ml of LB broth by vortex mixing. Bacterial suspension was diluted serially in saline and viable bacteria were counted by plating on LSW^-^ agar plates. The results were expressed as percentage of viable bacteria that survived the PB or PB plus HQ17-2 treatment versus the untreated (solvent) control.

### Preparation and Analysis of LPS

One hundred microliters of an overnight LB broth culture was inoculated onto LB agar plates with or without HQ17-2 and the plates were incubated for 6 h at 37°C. LPS extraction and analysis were performed as described previously [25].

### Binding of PB by LPS

The experiments for determining the binding of PB by LPS were performed as described previously [25], except that final concentration of 7.5, 15, 30 and 60 µg/ml of PB were used in the PB-binding reaction.

## Results

### Inhibition of Swarming in *P. mirabilis* by HQ17-2

**Figure 1:**
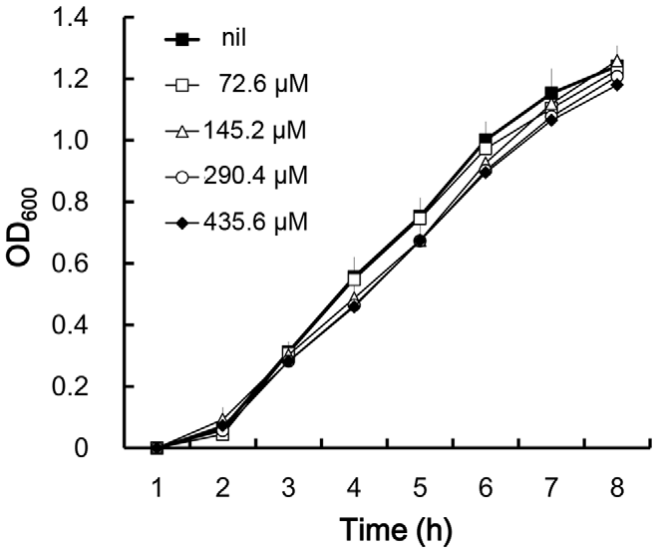
The effect of HQ17-2 on the growth of *P. mirabilis*. The overnight bacterial culture of the wild-type was diluted 1 : 100 into fresh LB broth containing different concentrations of HQ17-2 (0, 72.6, 145.2, 290.4 and 435.6 µM). The bacterial growth was monitored thereafter as OD_600_. Data are the mean of three determinations with standard deviations.

**Figure 2:**
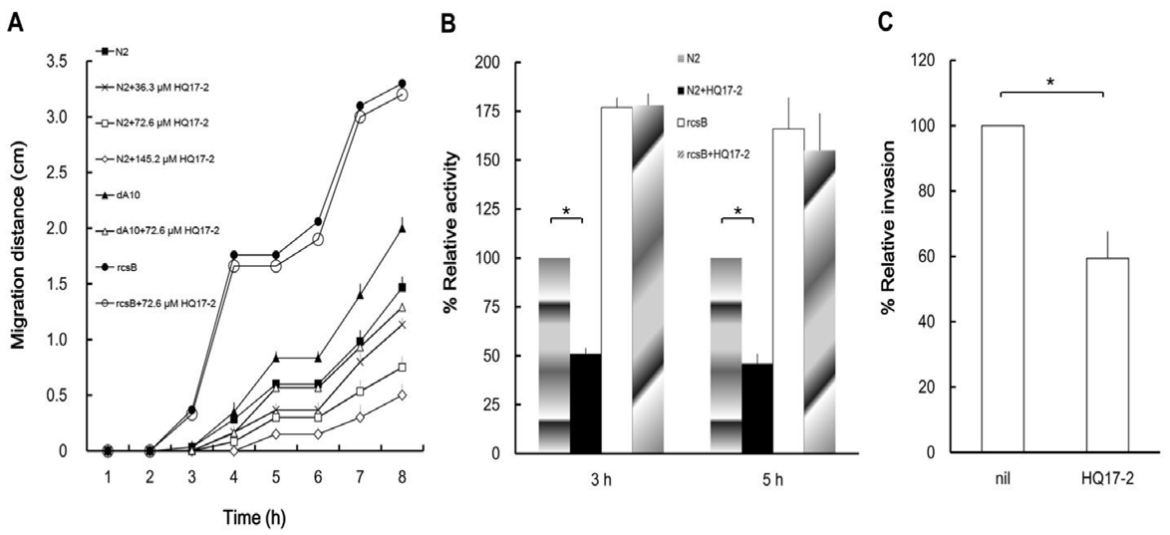
The effect of HQ17-2 on swarming motility, hemolysin activity and cell invasion ability of *P. mirabilis.* **(A)** HQ17-2 at 36.3, 72.6 or 145.2 µM was used to test the effect of HQ17-2 on swarming motility of the wild-type *P. mirabilis* (N2). Swarming motility of *rppA* (dA10) and *rcsB* (rcsB) mutants in the presence or absence of 72.6 µM HQ17-2 was also monitored. An aliquot (5 µl) of overnight cultures was inoculated onto the centers of LB swarming plates and the migration distance was measured hourly after inoculation. **(B)** Hemolysin activity of wild-type *P. mirabilis* and the *rcsB* mutant in the presence or absence of 72.6 µM HQ17-2. Hemolysin activity was determined at different time points after the bacteria were seeded onto LB agar plates. Only the results at 3 and 5 h after seeding are shown. **(C)** Invasion ability of wild-type *P. mirabilis* in the presence or absence of 72.6 µM HQ17-2. Data are the means of three independent experiments with standard deviations. In **B**, the value obtained from the wild-type in the absence of HQ17-2 was defined as 100% at 3 or 5 h, and all other values were expressed relative to this value. In **C**, the value obtained in the absence of HQ17-2 was defined as 100%, and the value in the presence of HQ17-2 was expressed relative to this value. In **B** and **C**, a significant difference was observed by Student’s *t-*test analysis (*, P<0.01**)**.

In the course of searching for compounds affecting swarming behavior of *P. mirabilis*, HQ17-2, a compound from the sap of the lacquer tree [28], at the concentration of 145.2 µM was found to inhibit swarming behavior of *P. mirabilis* on LB swarming agar plates (data not shown) at 37°C for 8 h. The effect of HQ17-2 on the growth of *P. mirabilis* was also tested. HQ17-2 at the concentrations up to 435.6 µM had no effect on the growth of *P. mirabilis* (Figure 1). To confirm the effect of HQ17-2 on *P. mirabilis* swarming, the bacteria were inoculated onto the centre of the swarming plates containing 36.3, 72.6 and 145.2 µM HQ17-2, and the migration distance of the bacteria was measured. As shown in Figure 2A, HQ17-2 dose-dependently inhibited swarming migration and could significantly inhibit swarming migration at the concentration as low as 36.3 µM. The HQ17-2-treated wild-type (at 36.3, 72.6 and 145.2 µM of HQ17-2) migrated slower than the untreated control at 4 h and thereafter till 8 h after inoculation (Figure 2A). These data indicate that HQ17-2 may be an anti-swarming agent for *P. mirabilis*. Since 72.6 µM HQ17-2 could effectively inhibit *P. mirabilis* swarming but had no cytotoxic effect on human urothelial cells (Figure S1), this concentration of HQ17-2 was used in the rest experiments of this study.

### Inhibition of Hemolysin Activity and Cell Invasion Ability of *P. mirabilis* by HQ17-2

In *P. mirabilis,* expression of virulence factors is regulated coordinately with swarming behavior [3], [5], [11]. Knowing that swarming was inhibited by HQ17-2 in *P. mirabilis*, we thus tested whether expression of virulence factors, including haemolysin and cell invasion, was also affected by HQ17-2. In the presence of HQ17-2, *P. mirabilis* N2 expressed significantly lower levels of haemolysin activity at 3, 4 and 5 h after inoculation (Figure 2B, only the results of 3 and 5 h were shown). The transcription of haemolysin gene, *hpmA*, was also inhibited by HQ17-2 (data not shown). Moreover, the cell invasion ability of *P. mirabilis* N2 was significantly lower in the presence of HQ17-2 than in the absence of HQ17-2 (Figure 2C).

### HQ17-2 Inhibited Swarming and Expression of *flhDC* through the RcsB Pathway in *P. mirabilis*

We have found that HQ17-2 could inhibit swarming in *P. mirabilis*. Since both RcsB and RppA pathways have been shown to be able to regulate swarming in *P. mirabilis* [5], [14], [25], [26], we thus tested whether these two pathways are involved in swarming inhibition ofHQ17-2. As shown in Figure 2A, while HQ17-2 could inhibit swarming in the wild-type and *rppA* mutant of *P. mirabilis*, HQ17-2 could not inhibit swarming in *rcsB* mutant. These data indicate that HQ17-2 may inhibit swarming through the RcsB-dependent pathway.

**Figure 3:**
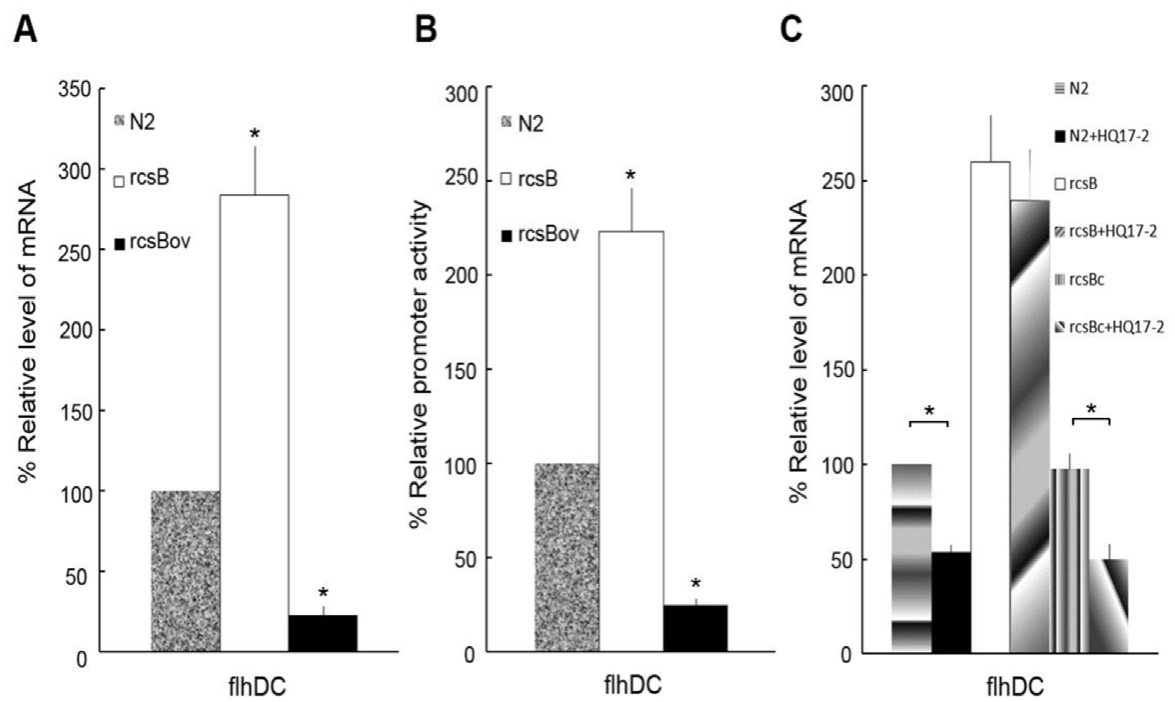
RcsB regulates *flhDC* expression and HQ17-2 inhibits *flhDC* expression in the wild-type and the *rcsB-*complemented *P. mirabilis* but not in the *rcsB* mutant. (**A**) and (**B**) The expression of *flhDC* in the wild type *P. mirabilis* (N2), the *rcsB* mutant (rcsB) and the *rcsB-*overexpressed strain (rcsBov). In **A**, the mRNA amount of *flhDC* was quantified by real-time RT-PCR at 4 h after inoculation. In **B**, the activities of XylE in the *flhDC*-*xylE* reporter plasmid-transformed N2, rcsB, and rcsBov strains were determined at 4 h after inoculation by the reporter assay. (**C**) The expression of *flhDC* mRNAs in wild-type *P. mirabilis*, the *rcsB* mutant and the *rcsB*-complemented strain in the presence or absence of 72.6 µM HQ17-2. The mRNA amount of *flhDC* was quantified by real-time RT-PCR after incubation with HQ17-2 for 4 h. In **A** and **B**, the value obtained for the wild-type was defined as 100%, and other values were expressed relative to this value. *, a significant difference was observed (P<0.01) in comparing to the wild-type. In **C**, the value obtained for the wild-type cells in the absence of HQ17-2 was defined as 100%. All data represent the averages of three independent experiments with standard deviations. A significant difference was observed by Student’s *t-*test analysis (*, P<0.01).

Previous studies have shown that the expression of *flhDC* gene is required for swarming and virulence factor expression [7], [9], [14], [17] and that RcsB can negatively regulate the expression of the *flhDC* gene in *E. coli*, *Salmonella* and *P. mirabilis* [14], [15], [16]. We thus first confirmed whether RcsB could do the same in *P. mirabilis* N2 strain. Our data indicated that the *flhDC* mRNA expression was increased in the *rcsB* mutant but was reduced in the *rcsB*-overexpressed strain (Figure 3A), indicating that RcsB can inhibit the expression of *flhDC* in *P. mirabilis* N2. This conclusion was also supported by the observation that the promoter activity of *flhDC* gene was increased in the *rscB* mutant but was decreased in the *rcsB*-overexpressed strain (Figure 3B). Together, these data demonstrate that RcsB can negatively regulate the expression of *flhDC* gene, resulting in reduced swarming motility in *P. mirabilis*.

Next, we tested whether HQ17-2 could inhibit the expression of *flhDC* gene in *P. mirabili*s and if yes, whether this inhibition was mediated through the RcsB- dependent pathway. As shown in Figure 3C, HQ17-2 could significantly inhibit the expression of *flhDC* mRNAs in the wild-type and the *rcsB*-complemented strain (rcsBc) but not in the *rcsB* mutant (not statistically significant), indicating that HQ17-2 may inhibit the expression of *flhDC* gene through the RcsB-dependent pathway.

The RcsB protein has been shown to repress *rcsB* gene expression by binding directly to the *rcsDB* promoter, negatively autoregulating the Rcs system [37]. To confirm HQ17-2 can activate the RcsB pathway, we tested the expression of *rcsB* in the presence of HQ17-2. By real-time RT-PCR, we indeed found that HQ17-2 downregulated *rcsB* expression probably due to the activation of RcsB pathway by HQ17-2, then resulting in negative autoregulation of RcsB (Fig. S2). The result supports that the negative autoregulation of RcsB may exist in *P. mirabili*s. In summary, HQ17-2 somehow activates RcsB, then leading to decreased expression of *rcsB* and *flhDC*, two genes negatively regulated by RcsB.

### HQ17-2 Increased Susceptibility of *P. mirabilis* to PB

It has been reported that increased expression of the *pmrHFIJKLM* operon, which confers PB resistance, was observed in *Salmonella* swarm cells and that *Salmonella* swarm cells exhibited greater tolerance to PB [38]. Considering the fact that HQ17-2 could inhibit swarming, we thus tested if PB susceptibility of *P. mirabilis* is altered by HQ17-2. As shown in Table 3, in the presence of 72.6 µM and 145.2 µM HQ17-2, PB MIC of wild-type *P. mirabilis*, which is highly resistant to PB [25], was decreased from over 40960 to 5120 µg/ml and 2560 µg/ml, respectively. Moreover, the checkerboard assay showed the synergy of HQ17-2 and PB at the combinations used (Figure S3).

**Table 3:** The effect of HQ17-2 on PB MIC levels of different *P. mirabilis* strains.

	MIC (µg/ml)	
Strain	without HQ17-2	with HQ17-2*
N2	>40960	5120
N2	>40960	2560#
dA10	256	256
dA10c	>40960	5120
dIp	4	4
dIpc	>40960	5120
rcsB	>40960	5120

* 72.6 µM HQ17-2;

# 145.2 µM HQ17-2; N2, wild-type; dA10, *rppA* mutant; dA10c, *rppA*-complemented strain; dIp, *pmrI* knockout mutant; dIpc, *pmrI*-complemented strain; rcsB, *rcsB* mutant.

To confirm the effect of HQ17-2 on the susceptibility of *P. mirabilis* to PB, we test the effect of HQ17-2 on 11 *P. mirabilis* clinical isolates. All isolates showed a decreased MIC level in the presence of HQ17-2 (Table 4).

**Table 4:** The effect of HQ17-2 on PB MIC levels of *P. mirabilis* clinical isolates.

	MIC (µg/ml)	
Isolate	without HQ17-2	with HQ17-2*
CI1	>40960	10240
CI2	>40960	5120
CI3	>40960	5120
CI4	>40960	10240
CI5	>40960	10240
CI6	>40960	5120
CI7	>40960	5120
CI8	>40960	5120
CI9	>40960	5120
CI10	>40960	5120
CI11	>40960	10240

* 72.6 µM HQ17-2.

**Figure 4:**
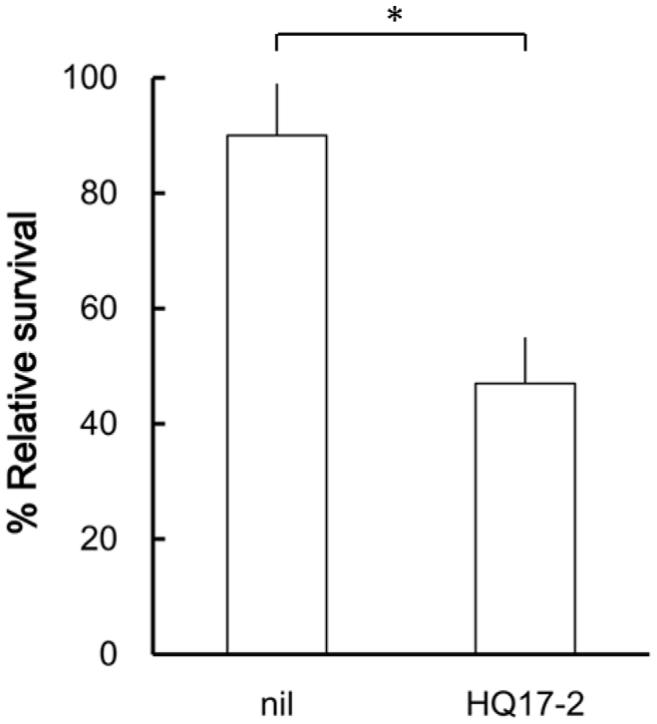
Killing of biofilm-grown *P. mirabilis* by PB in the presence or absence of HQ17-2. After biofilm formation of *P. mirabilis* on NC membranes, the membranes were applied with 10240 µg/ml PB plus 72.6 µM HQ17-2 or not. After incubation for 16 h, the number of viable bacteria of the individual membrane was determined. The results were expressed as percentage of viable bacteria that survived the PB or PB plus HQ17-2 treatment versus the control (i.e., not treated with PB and HQ17-2). The data are the averages and standard deviations of three independent experiments. A significant difference was observed by Student’s *t-*test analysis (*, P<0.01**)**.

An important cause of antibiotic failure in many infections is the formation of biofilms by infecting organisms. Biofilm formation produces phenotypic resistance even when bacteria are inherently antibiotic-sensitive [39]. We thus investigated the effect of HQ17-2 on the killing of biofilm-grown bacterial cells by PB using a model in which biofilms are formed on permeable membranes over LSW^-^ agar plates [36]. After biofilm formation, bacterial cells in the membranes were challenged with PB alone or PB plus HQ17-2. The survived bacteria on the membranes were determined after a further incubation of 16 h. We found PB (at 10240 or 20480 µg/ml) killed significantly more bacteria in the presence of HQ17-2 than in the absence of HQ17-2. The killing result of 10240 µg/ml PB in combination with HQ17-2 at 72.6 µM was shown in Figure 4.

Together, these data suggest that HQ17-2 can enhance PB susceptibility of laboratory and clinical isolates, as well as increase the efficacy of PB against biofilm-grown bacteria.

### MICs of Gentamycin, Kanamycin, Streptomycin, Ampicillin, Tetracycline, Ciprofloxacin and SDS were not Altered by HQ17-2

Knowing that HQ17-2 can enhance susceptibility of *P. mirabilis* to PB, we tested if HQ17-2 has the same effect on *P. mirabilis* to other antibiotics including gentamycin, kanamycin, streptomycin, ampicillin, tetracycline, and ciprofloxacin. No effect was observed on *P. mirabilis* susceptibility to gentamycin, kanamycin, streptomycin, ampicillin, tetracycline, and ciprofloxacin at the HQ17-2 concentration used, 72.6 or 145.2 µM (Table 5). We further examine the cell permeability of the HQ17-2 treated and untreated *P. mirabilis* by the SDS susceptibility assay and the crystal violet uptake assay. We found HQ17-2 doesn’t cause changes of cell permeability (Table 5 and our unpublished data). Thus, other mechanisms exist to render the HQ17-2 treated *P. mirabilis* more susceptible to PB.

**Table 5:** The effect of HQ17-2 on MICs of SDS and various antimicrobial agents.

	MIC (µg/ml)		
	nil	HQ17-2 (µM)	
		72.6	145.2
gentamycin	4	4	4
kanamycin	16	16	16
streptomycin	32	32	32
tetracycline	32	32	32
ampicillin	16	16	16
ciprofloxacin	2^−6^	2^−5^	2^−5^
SDS	0.4%	0.4%	0.4%

### HQ17-2 Inhibited Promoter Activities of *rppA* and *pmrI* in *P. mirabilis*

Previously we reported that the RppA signaling pathway positively regulates PB resistance [25] and that PmrI, whose expression is induced by RppA, is involved in LPS modification and positively correlated to PB resistance in *P. mirabilis* [26]. To test if the RppA signaling pathway is involved in HQ17-2-mediated enhanced susceptibility of *P. mirabilis* to PB, the effect of HQ17-2 on susceptibility of the *rppA* and *pmrI* mutants to PB was assessed. HQ17-2 reduced PB MIC of the wild-type but not that of the *rppA* and *pmrI* mutants (Table 3). The *rppA* and *pmrI*-complemented strains restored reduced PB MIC in the presence of HQ17-2. These indicate that the RppA pathway is involved in HQ17-2-mediated enhancement of PB susceptibility in *P. mirabilis*.

**Figure 5:**
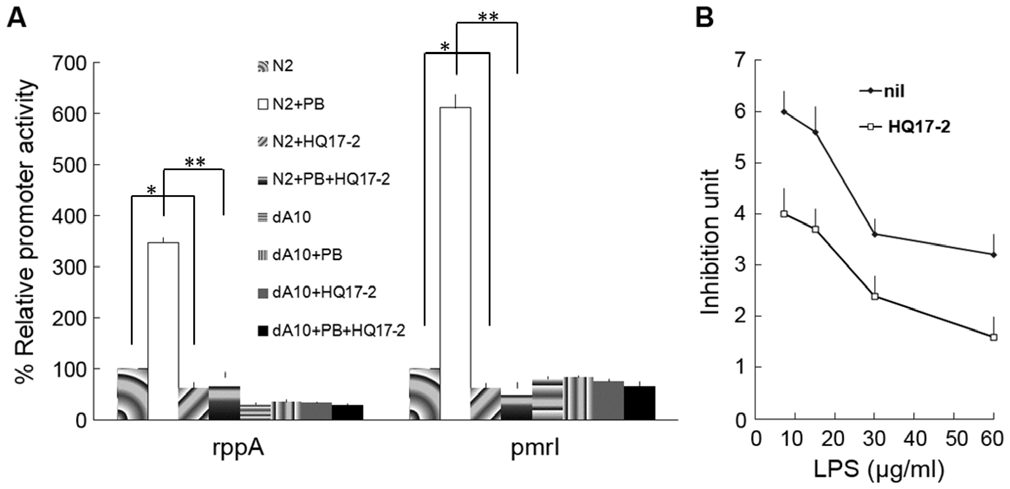
The effect of HQ17-2 on the promoter activities of *rppA* and *pmrI* genes and on the PB-binding ability of LPS in *P. mirabilis.* (**A**) The effect of HQ17-2 on the promoter activities of the *rppA* and *pmrI* in a noninduced or the PB-induced condition in wild-type *P. mirabilis* and the *rppA* mutant. Wild-type *P. mirabilis* (N2) and *rppA* mutant (dA10) were treated with 72.6 µM HQ17-2 or not in a noninduced or the PB-induced condition (1 µg/ml PB) and *rppA* and *pmrI* reporter assays were performed at 4 h after inoculation as described in the Materials and Methods. The value obtained for the wild-type in the absence of HQ17-2 and PB was defined as 100%, and other values were expressed relative to this value. A significant difference was observed by Student’s *t-*test analysis (*, P<0.05; **, P<0.01). (**B**) The effect of HQ17-2 on the PB-binding ability of LPS. PB-binding ability of LPS purified from *P. mirabilis* treated with HQ17-2 (72.6 µM) or not was determined. Various amounts of purified LPS were subjected to the PB-binding assay and the unbound PB was then subjected to the *E. coli* inhibition assay. The data are the averages of three independent experiments with standard deviations.

To further confirm this, reporter assays using *rppA* or *pmrI* promoter fused with *xylE* gene were performed. The promoter activity of both *rppA* and *pmrI* genes was lower in the presence of HQ17-2 than in the absence of HQ17-2 in wild-type *P. mirabilis* but HQ17-2 has no effect on the promoter activity of both *rppA* and *pmrI* in the *rppA* mutant (Figure 5A). We found that the *rppA* mutant still has low level of promoter activity, indicating *rppA* promoter is also subject to controls by regulators other than RppA in *P. mirabilis*. Because of the low basal level of *pmrI* expression in the wild-type and the *rppA* mutant in the absence of HQ17-2, corresponding to a noninduced condition of the RppA
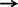
PmrI pathway [26], we tested the effect of HQ17-2 on the expression of *rppA* and *pmrI* (an indicator for activation of the RppA-PmrI pathway) of the wild-type and the *rppA* mutant in an induced condition (in the presence of 1 µg/ml PB). We found that HQ17-2 can suppress much more significantly the expression of *rppA* and *pmrI* in the wild-type but not in the *rppA* mutant in the induced condition of the RppA
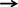
PmrI pathway (Figure 5A).

In summary, these data suggest that HQ17-2 enhances PB susceptibility by inhibiting RppA
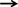
PmrI pathway, which leads to inhibition of RppA expression (due to autopositive regulation by itself [25]) and in turn the expression of PmrI, a protein involved in modification of LPS and modulation of PB susceptibility [26].

Knowing that HQ17-2 can modulate the RppA
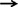
PmrI pathway, which is known to be involved in LPS modification [26], we next tested whether HQ17-2 treatment could induce alterations of LPS in *P. mirabilis*. No significant difference in the level of LPS and the LPS SDS-PAGE profile was observed between HQ17-2-treated and untreated cells (data not shown). However, LPS purified from HQ17-2-treated cells bound a larger amount of PB than that purified from untreated cells (Figure 5B), indicating that there is a qualitative change in the LPS of HQ17-2-treated *P. mirabilis* and this change causes LPS to have higher binding activity to PB. The Oregon Green 514 polymyxin B (PB-OG) binding assay of whole bacteria or LPS (a more direct experiment) was also performed to confirm the PB binding ability of LPS from HQ17-2-treated or not cells. Figure S4 showed that HQ17-2-treated *P. mirabilis* and LPS from the treated cells bind more PB-OG than the untreated control. Therefore, HQ17-2 treatment causes *P. mirabilis* to have higher affinity for PB, which in turn makes cells become more sensitive to PB.

## Discussion

The emergence of multiple drug-resistant bacterial infections poses a major threat to public health. As a consequence, there are renewed concepts in antibacterial strategies, among them, strategies to attenuate bacterial virulence have attracted most attention [31]. The facts that bacterial virulence is regulated by TCSs and that TCSs are widely distributed in bacteria but not in human have established the TCSs as attractive targets for antimicrobial agents [32]. In this study, for the first time, we demonstrate that HQ17-2, a natural compound from the lacquer tree, can inhibit hemolysin activity and swarming motility of *P. mirabilis* through modulating the RcsB pathway (Figure 2, 3) and increase PB susceptibility in *P. mirabilis* through modulating the RppAB TCS (Table 3). This ability to reduce virulence factor expression and increase drug susceptibility in *P. mirabilis* makes HQ17-2 become a potential therapeutic agent to control *P. mirabilis* infections.

The activation of the RcsB pathway is known to inhibit swarming motility and hemolysin expression in *P. mirabilis* [6], [7], [9], [14], [40]. We found that HQ17-2 had a significant inhibitory effect on the swarming motility and hemolysin activity of the wild-type but not the *rcsB* mutant of *P. mirabilis* (Figure 2A & 2B). Moreover, HQ17-2 inhibited the expression of *flhDC* gene, a downstream gene involved in swarming and negatively regulated by RcsB [14], [15], [16], in the wild-type and the *rcsB*-complemented strain but not in the *rcsB* mutant of *P. mirabilis* (Figure 3). These results suggest that HQ17-2 inhibit swarming motility and hemolysin activity of *P. mirabilis* through the RcsB-dependent pathway and that HQ17-2 can serve as an activator of the RcsB pathway. Besides, we found that HQ17-2 downregulated *rcsB* expression probably due to the activation of RcsB pathway by HQ17-2, then resulting in negative autoregulation of RcsB (Fig. S2). Altogether, HQ17-2 somehow activates the RcsB pathway, then leading to decreased expression of *rcsB* and *flhDC*, two genes negatively regulated by RcsB in *P. mirabilis*. How could HQ17-2 activate the RcsB pathway? Hydroquinone is known to be able to influence the structure or topology of the cell membrane [41] and perturbation of cell membrane has been shown to activate the RcsB pathway [12]. HQ17-2, a kind of hydroquinones, may perturb cell membrane, which in turn leads to activation of the RcsB pathway, resulting in inhibition of swarming and hemolysin expression in *P. mirabilis*. Though the underlying mechanism is not clear, the finding that an integral membrane protein is capable of mediating induction of gene expression by hydroquinone [42] further supports the HQ17-2-mediated activation of RcsB pathway.

The unaltered *P. mirabilis* susceptibility to gentamycin, kanamycin, streptomycin, ampicillin, tetracycline, ciprofloxacin and SDS in the presence of HQ17-2 at the concentrations used indicates that HQ17-2 affects PB susceptibility through a specific mechanism. We found that HQ17-2 could increase PB susceptibility in the wild-type but not in the *rppA* and *pmrI* mutants of *P. mirabilis* (Table 3), suggesting that HQ17-2 increase PB susceptibility through the RppA
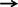
PmrI dependent pathway. HQ17-2 shares structural similarity with the published inhibitor of VirAG TCS [43]. Through binding to the VirA sensor, the VirAG inhibitor competes with phenol, which is the inducer of VirAG TCS [43]. Whether HQ17-2 could act as the RppAB inhibitor by targeting RppB sensor to interfere with autophosphorylation and phosphotransfer to RppA is not known. Alternatively, HQ17-2 could target RppA response regulator directly to inhibit RppA’s promoter-binding or transcription-activating activity. Experiments aiming to resolve these questions are in progress.

It is of interest to note that RppA is a negative regulator of swarming and hemolysin expression in *P. mirabilis* [25]. In this study, we demonstrated that HQ17-2 could increase PB susceptibility presumably through inhibiting the RppA pathway. Inhibition of the RppA pathway by HQ17-2 theoretically should lead to activation of swarming and hemolysin expression. Why HQ17-2 causes inhibition of swarming and hemolysin expression instead? One possible explanation is that swarming motility and hemolysin expression is regulated primarily through the RcsB pathway in *P. mirabilis*. This argument is supported by the following observations: (i) the *rcsB* mutant migrates much faster than the *rppA* mutant (Figure 1A), and (ii) HQ17-2 could not inhibit swarming in *rcsB* mutant but could do so effectively in the *rppA* mutant (Figure 1A). The other explanation may be the preference of HQ17-2 for the RcsB pathway.

In this report, we demonstrate that HQ17-2 can inhibit swarming ability, hemolysin activity, and cell invasion ability of *P. mirabilis*. Moreover, we show that HQ17-2 can increase PB susceptibility in laboratory strain and clinical isolates of *P. mirabilis*. Our unpublished data also revealed that HQ17-2 could inhibit tyrosinase activity of *P. mirabilis*, which in turn caused inhibition of melanin production, leading to a reduced survival of *P. mirabilis* upon H_2_O_2_ exposure (data not shown). In addition to these anti-bacterial effects, we also found that HQ17-2 in combination with urea (the major component in human urine) exhibited synergistic killing effect on *P. mirabilis* (Figure S5), even though HQ17-2 alone had no killing effect and urea alone even had growth-promoting effect on *P. mirabilis* at the concentrations used (Figure S5). Although the mechanism underlying the synergistic killing effect of HQ17-2 and urea is not clear, it is tempting to apply HQ17-2 onto urinary catheters to prevent *P. mirabilis* infections. Together, our data not only identify HQ17-2 as a potential anti-*P mirabilis* agent but also provide a new possible way, namely coating urinary catheters with HQ17-2, to prevent catheter-derived *P. mirabilis* infection.

## Supporting Information

Figure S1 **Cell viability assay.** Cell viability was evaluated by measuring cellular acid phosphatase (ACP) activity (J Agric Food Chem 2009, 57: 2200–2205). Briefly, 4×10^3^ human urothelial NTUB1 cells (Antimicrob Agents Chemother 2010, 54: 1564–1571) in 180 µl of RPMI 1640 medium were cultured in 96-well plates and treated with various concentrations of HQ-172 for 48 hours. After that, the cells were washed twice with PBS and incubated with a 100-µl assay buffer containing 0.1 M sodium acetate, 0.1% Triton X-100, and 10 mM 4-nitrophenyl phosphate. After incubation at 37°C for 30 minutes, the reaction was stopped by addition of a 10-µl 1 N NaOH, and OD_405_ was measured using a microplate reader (Molecular Devices).
(TIF)

Figure S2 **HQ17-2 downregulates** ***rcsB*** **expression.** The real-time RT-PCR was performed as described in the Materials and Methods except the primers used, rcsBrealtimeF (GCAGATGCTCTTATCACC) and rcsBrealtimeR (CAGGCGCACCTTGTTTTA). The levels of *rcsB* mRNAs were normalized against 16S rRNAs. The value obtained from cells without treatment with HQ17-2 was set at 100%.
(TIF)

Figure S3 **The checkerboard method showing the synergy of HQ17-2 and polymyxin B (PB).** A broth microdilution checkerboard method was used to determine the susceptibility of *P. mirabilis* to the indicated combinations of PB and HQ17-2. The stock solutions and serial twofold dilutions of each drug were prepared prior to testing. A total of 100 µl of Mueller-Hinton broth was distributed into each well of the microtiter plates. PB was serially diluted along the ordinate, while HQ17-2 was diluted along the abscissa. Each microtiter well was inoculated with 100 µl of a *P. mirabilis* inoculum of 5×10^5^ CFU/ml, and the plates were incubated at 35°C for 18 h under aerobic conditions. The assays were performed in triplicate for each combination. Dark areas indicate visible growth. The ΣFICs in the indicated combinations are as follows: *, (10240/over 40960)+(36.3/over 580.8) <0.31 #, (5120/over 40960)+(72.6/over 580.8) <0.25 &, (2560/over 40960)+(145.2/over 580.8) <0.31.
(TIF)

Figure S4 **The effect of HQ17-2 on the binding of** ***P. mirabilis*** **with fluorescent polymyxin B. (A)** The effect of HQ17-2 on the binding of *P. mirabilis* whole cells with fluorescent polymyxin B. Wild-type *P. mirabilis* was treated with 72.6 µM HQ17-2 or not for 5 h before incubation for 10 min with Oregon Green 514 polymyxin B (PB-OG) (Invitrogen) at the indicated concentrations (PLoS Pathogens 2011, 7: e1002454). After washing, the cells were resuspended in PBS and placed into 96-well plates for analysis. Fluorescence (480 nm excitation and 535 nm emission) and OD_600_ of each well was determined using a microplate reader. Each experiment was repeated in triplicate and data reported as a ratio of fluorescence intensity to OD_600_. The HQ17-2 treated cells show increased binding of PB-OG when compared to the untreated control. An asterisk is used to indicate data points that are significantly different from that of the untreated control (p<0.05). **(B)** The effect of HQ17-2 on the binding of *P. mirabilis* LPS with fluorescent polymyxin B. Aliquots of purified LPS from the cells treated with 72.6 µM HQ17-2 or not were diluted to final concentrations of 15, 30, 60, 100, 150 and 300 µg/ml with a 2 mM HEPES (pH 7.2) solution in microplate wells. PB-OG was added to the LPS solutions to obtain the concentration of 5 µg/ml and then the solutions were incubated at 37°C for 30 min. After incubation, the solutions were centrifuged (12000 *g*, 10 min), the supernatants were discarded and the PB-OG bound LPS was resuspended in a 100-µl HEPES solution. Fluorescence was determined. Each experiment was repeated in duplicate. The fluorescence level of PB-OG bound by 300 µg/ml LPS from the HQ17-2 treated cells was set to 100% and other data were relative to this value.
(TIF)

Figure S5 **The synergistic killing effect of urea and HQ17-2.** An overnight culture of *P. mirabilis* was diluted 100-fold and incubated for 3 h before the assay. A 0.5 ml bacterial solution (1.5×10^8^ CFU/ml) was inoculated into 0.45 ml N-minimal medium (5 mM KCl, 7.5 mM (NH_4_)_2_SO_4_, 0.5 mM K_2_SO_4_, 1 mM KH_2_PO_4_, 0.1 mM Tris-HCl, 0.2% glucose, 0.01% casamino acids, PH7.4) with urea and HQ17-2 in different combination as shown. After incubation at 37°C for 1 h and 4 h, the viable bacterial count was determined by plating on LSW^-^ agar plates. The results were expressed as percentage of viable bacteria that survived the urea or urea plus HQ17-2 treatment versus the untreated control (no urea or HQ17-2). **(A)** HQ17-2 at 36.3, 72.6 or 145.2 µM in combination with 150 mM urea inhibited the growth of *P. mirabilis*. *, a significant difference was observed in comparing with the survival in the N-minimal medium containing urea only by Student’s *t-*test analysis (P< 0.01**)**. **(B)** Urea at 75, 150 or 300 mM in combination with 72.6 µM HQ17-2 decreased *P. mirabilis* survival. A significant difference was observed by Student’s *t-*test analysis (*, P<0.01**)**.
(TIF)
