# Supplementary figures and images for: 10′(Z),13′(E)-Heptadecadienylhydroquinone Inhibits Swarming and Virulence Factors and Increases Polymyxin B Susceptibility in Proteus mirabilis
Source: PLoS One. 2012 Sep 20;7(9):e45563. doi: 10.1371/journal.pone.0045563 (PMC3447793; doi:10.1371/journal.pone.0045563)

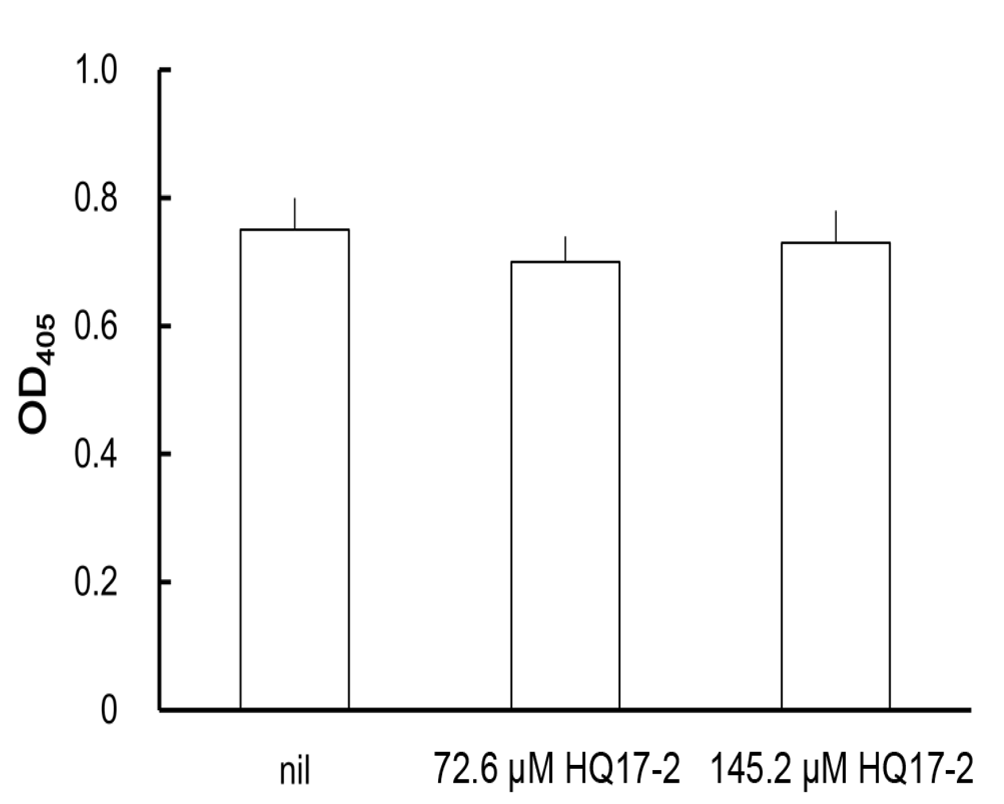

Supplement: Figure S1 — Cell viability assay. Cell viability was evaluated by measuring cellular acid phosphatase (ACP) activity (J Agric Food Chem 2009, 57: 2200–2205). Briefly, 4×10^3^ human urothelial NTUB1 cells (Antimicrob Agents Chemother 2010, 54: 1564–1571) in 180 µl of RPMI 1640 medium were cultured in 96-well plates and treated with various concentrations of HQ-172 for 48 hours. After that, the cells were washed twice with PBS and incubated with a 100-µl assay buffer containing 0.1 M sodium acetate, 0.1% Triton X-100, and 10 mM 4-nitrophenyl phosphate. After incubation at 37°C for 30 minutes, the reaction was stopped by addition of a 10-µl 1 N NaOH, and OD405 was measured using a microplate reader (Molecular Devices). (TIF) [file pone.0045563.s001.tif]

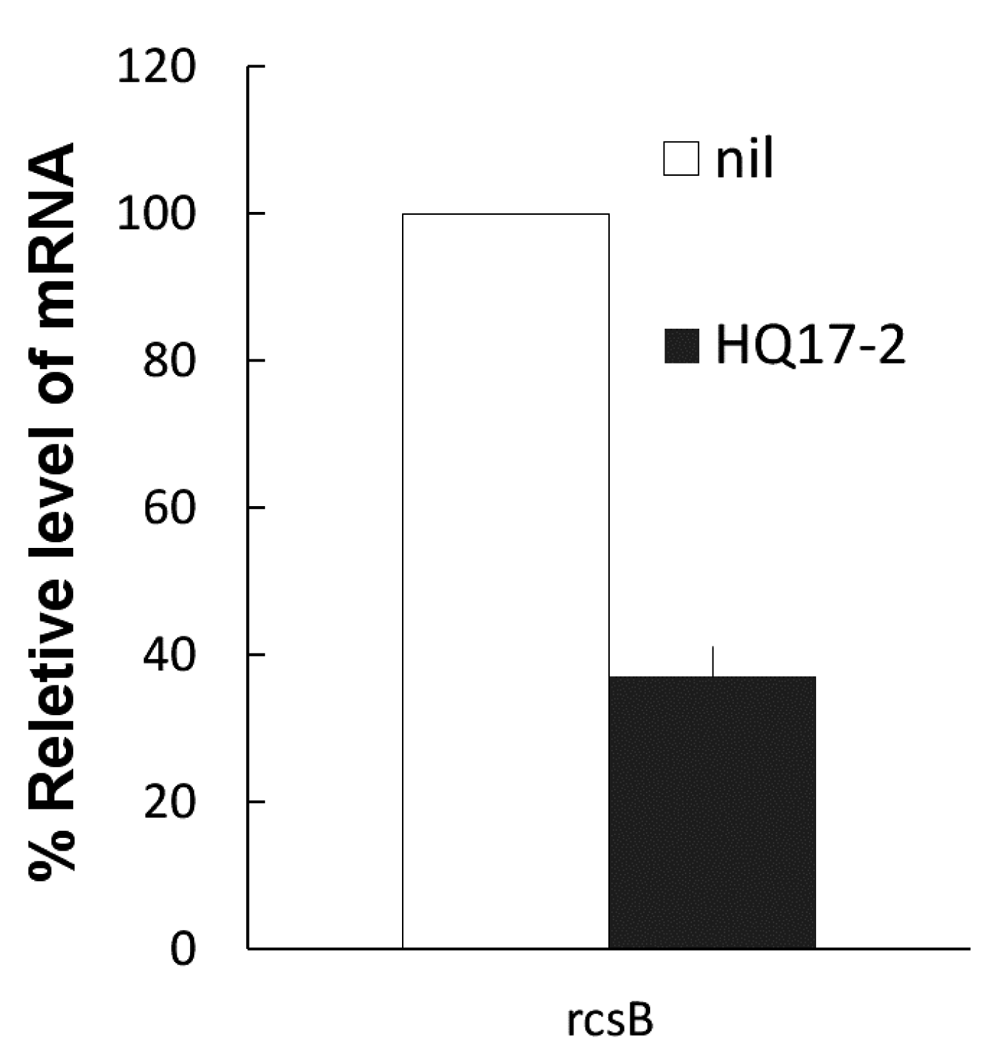

Supplement: Figure S2 — HQ17-2 downregulates rcsB expression. The real-time RT-PCR was performed as described in the Materials and Methods except the primers used, rcsBrealtimeF (GCAGATGCTCTTATCACC) and rcsBrealtimeR (CAGGCGCACCTTGTTTTA). The levels of rcsB mRNAs were normalized against 16S rRNAs. The value obtained from cells without treatment with HQ17-2 was set at 100%. (TIF) [file pone.0045563.s002.tif]

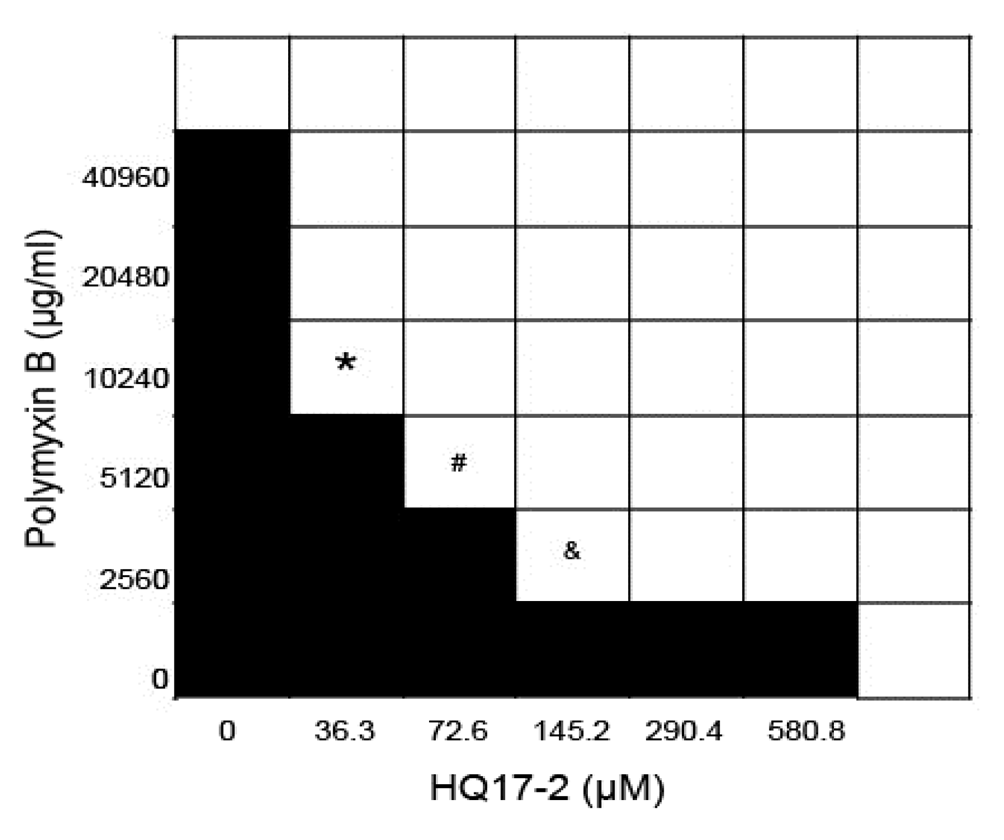

Supplement: Figure S3 — The checkerboard method showing the synergy of HQ17-2 and polymyxin B (PB). A broth microdilution checkerboard method was used to determine the susceptibility of P. mirabilis to the indicated combinations of PB and HQ17-2. The stock solutions and serial twofold dilutions of each drug were prepared prior to testing. A total of 100 µl of Mueller-Hinton broth was distributed into each well of the microtiter plates. PB was serially diluted along the ordinate, while HQ17-2 was diluted along the abscissa. Each microtiter well was inoculated with 100 µl of a P. mirabilis inoculum of 5×10^5^ CFU/ml, and the plates were incubated at 35°C for 18 h under aerobic conditions. The assays were performed in triplicate for each combination. Dark areas indicate visible growth. The ΣFICs in the indicated combinations are as follows: *, (10240/over 40960)+(36.3/over 580.8) <0.31 #, (5120/over 40960)+(72.6/over 580.8) <0.25 &, (2560/over 40960)+(145.2/over 580.8) <0.31. (TIF) [file pone.0045563.s003.tif]

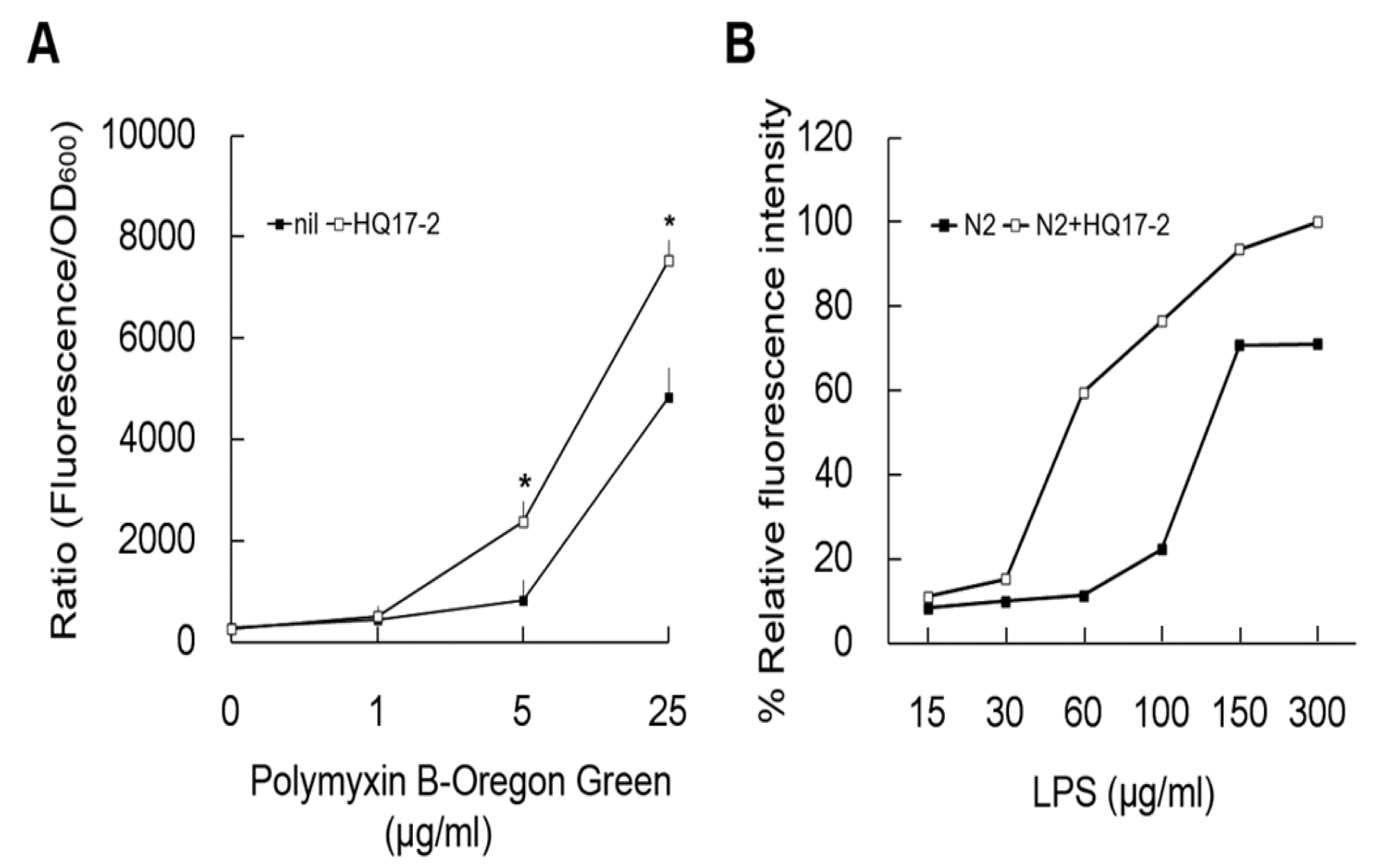

Supplement: Figure S4 — The effect of HQ17-2 on the binding of P. mirabilis with fluorescent polymyxin B. (A) The effect of HQ17-2 on the binding of P. mirabilis whole cells with fluorescent polymyxin B. Wild-type P. mirabilis was treated with 72.6 µM HQ17-2 or not for 5 h before incubation for 10 min with Oregon Green 514 polymyxin B (PB-OG) (Invitrogen) at the indicated concentrations (PLoS Pathogens 2011, 7: e1002454). After washing, the cells were resuspended in PBS and placed into 96-well plates for analysis. Fluorescence (480 nm excitation and 535 nm emission) and OD600 of each well was determined using a microplate reader. Each experiment was repeated in triplicate and data reported as a ratio of fluorescence intensity to OD600. The HQ17-2 treated cells show increased binding of PB-OG when compared to the untreated control. An asterisk is used to indicate data points that are significantly different from that of the untreated control (p<0.05). (B) The effect of HQ17-2 on the binding of P. mirabilis LPS with fluorescent polymyxin B. Aliquots of purified LPS from the cells treated with 72.6 µM HQ17-2 or not were diluted to final concentrations of 15, 30, 60, 100, 150 and 300 µg/ml with a 2 mM HEPES (pH 7.2) solution in microplate wells. PB-OG was added to the LPS solutions to obtain the concentration of 5 µg/ml and then the solutions were incubated at 37°C for 30 min. After incubation, the solutions were centrifuged (12000 g, 10 min), the supernatants were discarded and the PB-OG bound LPS was resuspended in a 100-µl HEPES solution. Fluorescence was determined. Each experiment was repeated in duplicate. The fluorescence level of PB-OG bound by 300 µg/ml LPS from the HQ17-2 treated cells was set to 100% and other data were relative to this value. (TIF) [file pone.0045563.s004.tif]

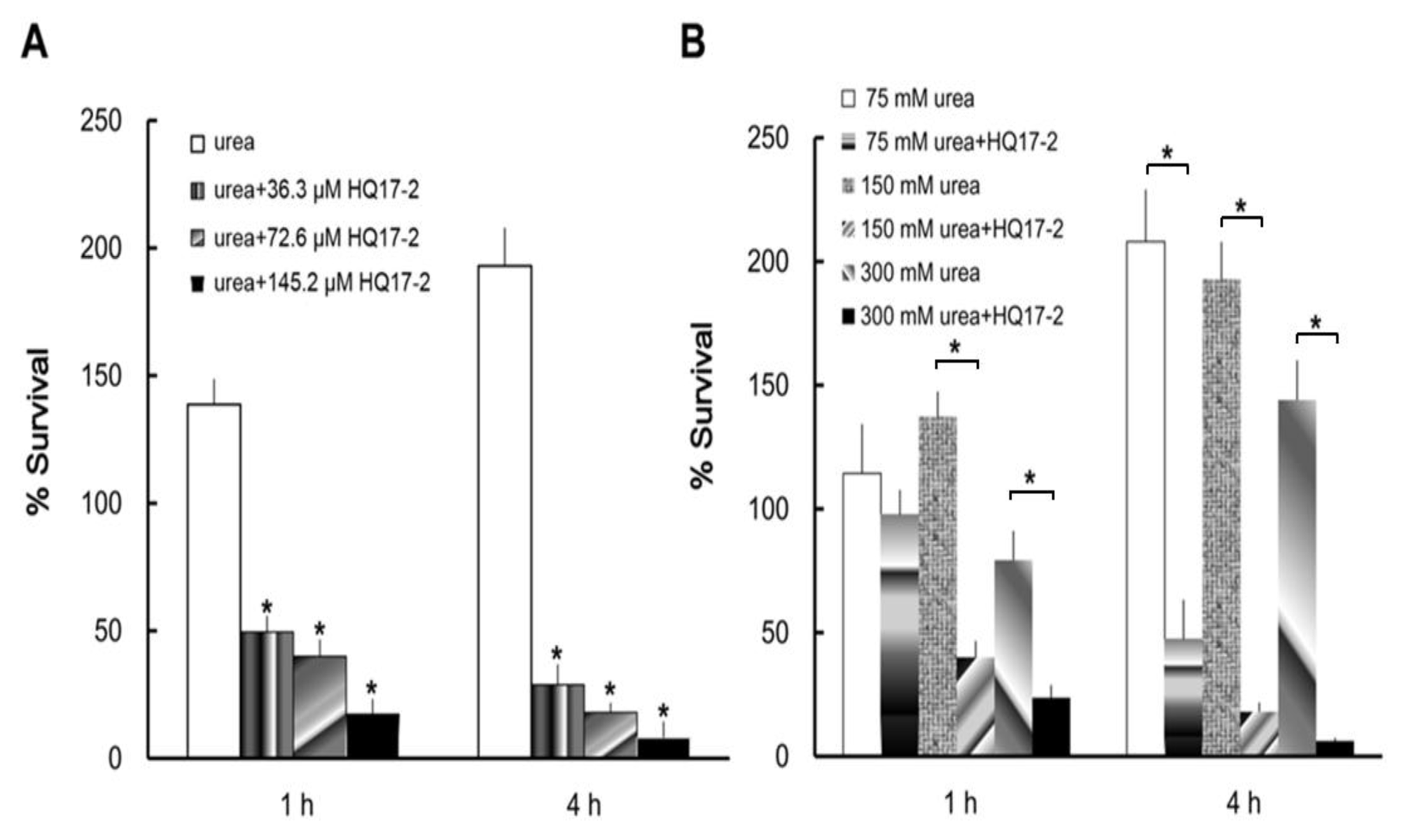

Supplement: Figure S5 — The synergistic killing effect of urea and HQ17-2. An overnight culture of P. mirabilis was diluted 100-fold and incubated for 3 h before the assay. A 0.5 ml bacterial solution (1.5×10^8^ CFU/ml) was inoculated into 0.45 ml N-minimal medium (5 mM KCl, 7.5 mM (NH4)2SO4, 0.5 mM K2SO4, 1 mM KH2PO4, 0.1 mM Tris-HCl, 0.2% glucose, 0.01% casamino acids, PH7.4) with urea and HQ17-2 in different combination as shown. After incubation at 37°C for 1 h and 4 h, the viable bacterial count was determined by plating on LSW- agar plates. The results were expressed as percentage of viable bacteria that survived the urea or urea plus HQ17-2 treatment versus the untreated control (no urea or HQ17-2). (A) HQ17-2 at 36.3, 72.6 or 145.2 µM in combination with 150 mM urea inhibited the growth of P. mirabilis. *, a significant difference was observed in comparing with the survival in the N-minimal medium containing urea only by Student’s t-test analysis (P< 0.01). (B) Urea at 75, 150 or 300 mM in combination with 72.6 µM HQ17-2 decreased P. mirabilis survival. A significant difference was observed by Student’s t-test analysis (*, P<0.01). (TIF) [file pone.0045563.s005.tif]
